# Supplementary material for: Cold and hot fibrosis define clinically distinct cardiac pathologies
Source: Cell Syst. 2025 Mar 19;16(3):None. doi: 10.1016/j.cels.2025.101198 (PMC11922821; doi:10.1016/j.cels.2025.101198)
Supplement: Document S1. Figures S1–S9, Note S1, and supplemental references [file mmc1.pdf]

Cell Systems, Volume *16*

Supplemental information

Cold and hot fibrosis define clinically distinct cardiac pathologies

Shoval Miyara, Miri Adler, Kfir B. Umansky, Daniel Häußler, Elad Bassat, Yalin Divinsky, Jacob Elkahal, David Kain, Daria Lendengolts, Ricardo O. Ramirez Flores, Hanna Bueno-Levy, Ofra Golani, Tali Shalit, Michael Gershovits, Eviatar Weizman, Alexander Genzelinakh, Danielle M. Kimchi, Avraham Shakked, Lingling Zhang, Jingkui Wang, Andrea Baehr, Zachary Petrover, Rachel Sarig, Tatjana Dorn, Alessandra Moretti, Julio Saez-Rodriguez, Christian Kupatt, Elly M. Tanaka, Ruslan Medzhitov, Achim Krüger, Avi Mayo, Uri Alon, and Eldad Tzahor

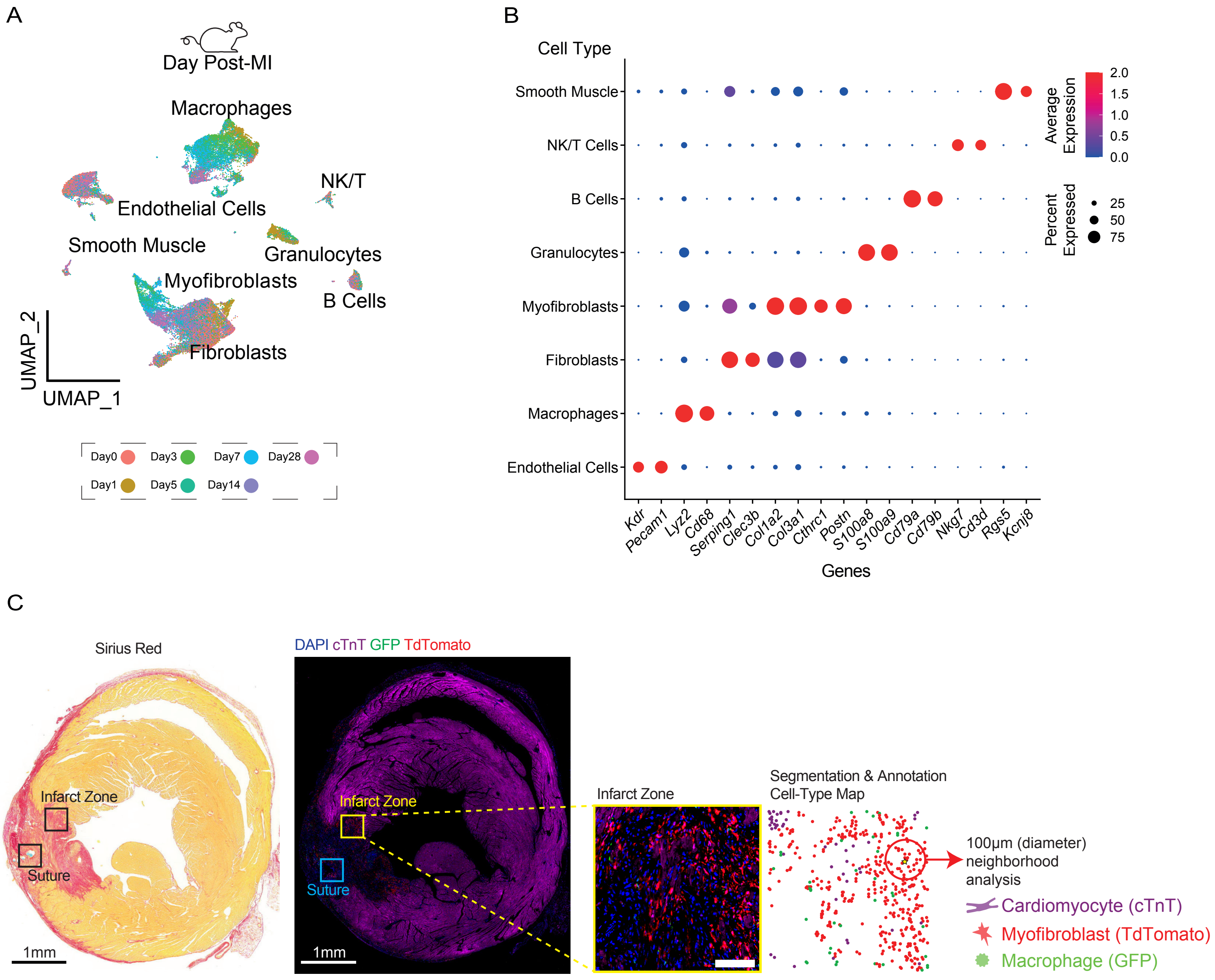
A
Day Post-MI
Macrophages
NK/T
Endothelial Cells
Smooth Muscle
Granulocytes
Myofibroblasts
B Cells
Fibroblasts
UMAP_2
UMAP_1
Day0
Day3
Day7
Day28
Day1
Day5
Day14
B
Cell Type
Smooth Muscle
NK/T Cells
B Cells
Granulocytes
Myofibroblasts
Fibroblasts
Macrophages
Endothelial Cells
Kdr
Pecam1
Lyz2
Cd68
Serping1
Clec3b
Col1a2
Col3a1
Cthrc1
Postn
S100a8
S100a9
Cd79a
Cd79b
Nkg7
Cd3d
Rgs5
Kcnj8
Genes
Average Expression
2.0
1.5
1.0
0.5
0.0
Percent Expressed
25
50
75
C
Sirius Red
Infarct Zone
Suture
1mm
DAPI cTnT GFP TdTomato
Infarct Zone
Suture
1mm
Infarct Zone
Segmentation & Annotation
Cell-Type Map
100µm (diameter)
neighborhood
analysis
Cardiomyocyte (cTnT)
Myofibroblast (TdTomato)
Macrophage (GFP)

Figure S1. Single-cell mRNA-sequencing, and spatial neighborhood analysis pipeline for the macrophage-myofibroblasts double reporter mice following MI, related to Figure 1

(A) Complementary scRNAseq data to Figure 1B. Total cardiac left ventricle interstitial cells following MI at days: 1 (yellow), 3 (light green), 5 (dark green), 7 (blue), 14 (purple) and 28 (pink). n = 1 per time-point.

(B) Known and top differentially expressed genes per cluster used for cluster annotation.

(C) Neighborhood analysis pipeline for double-reporter mice (MAMY) following MI. from left to right- annotation of immunofluorescence Infarct Zones and Suture were based on consecutive histological sections stained with sirius red. 400µm X 400µm fields of view (FOVs) were picked for each biological replicate. Cells were segmented (based on nuclear DAPI stain, blue) and annotated (STAR Methods) and a cell-type map was generated to represent three cell-types in their correct spatial localization: Macrophages (GFP+, green), myofibroblasts (TdTomato+, red) and cardiomyocytes (cTnT+, purple). For each cell-type identified the cell composition within its neighborhood: measured as a 50µm radius (100µm in diameter) from the center of each cell was determined. Both absolute counts and percentages of the three cell-types were determined.

A

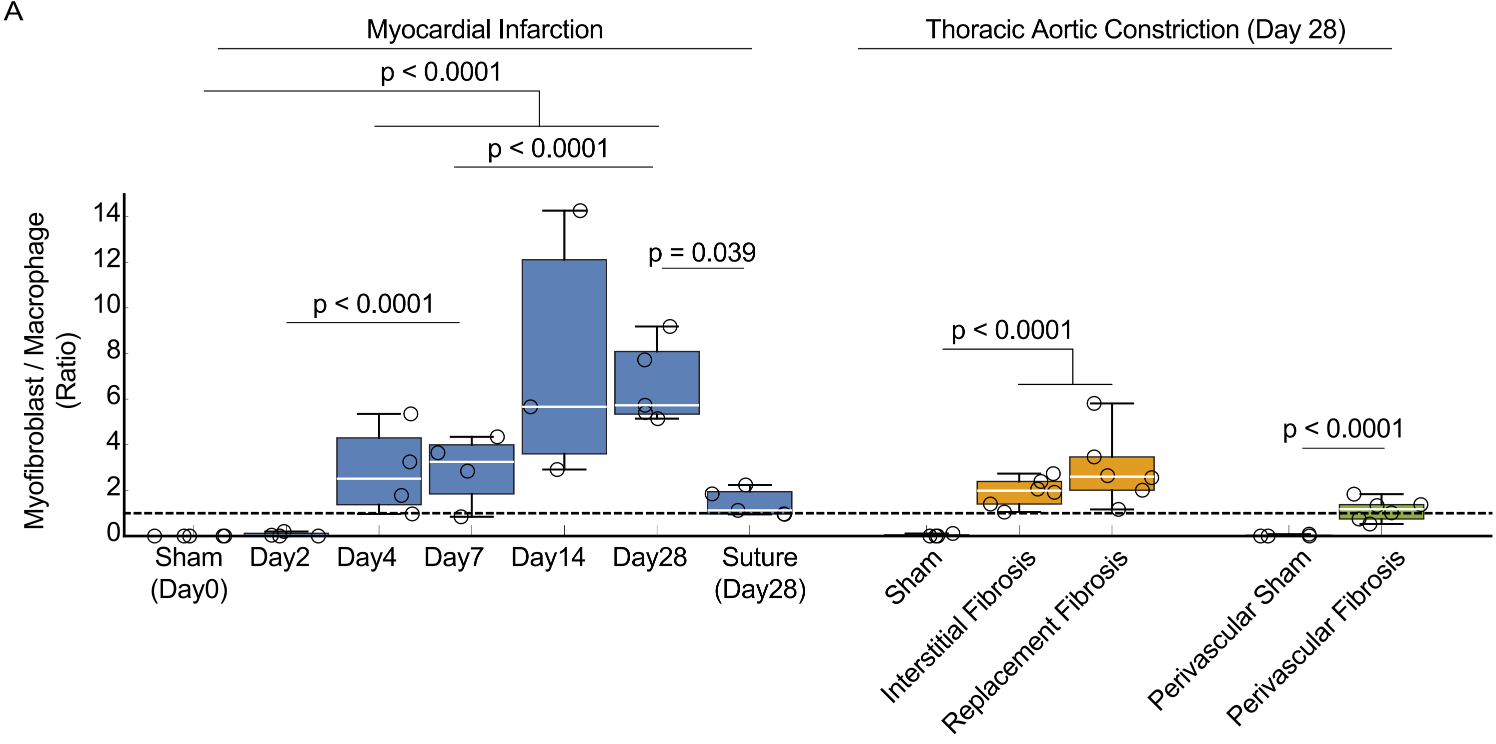

Myocardial Infarction
Thoracic Aortic Constriction (Day 28)
p < 0.0001
p < 0.0001
p = 0.039
p < 0.0001
p < 0.0001
p < 0.0001
Myofibroblast / Macrophage (Ratio)
0
2
4
6
8
10
12
14
Sham (Day0)
Day2
Day4
Day7
Day14
Day28
Suture (Day28)
Sham
Interstitial Fibrosis
Replacement Fibrosis
Perivascular Sham
Perivascular Fibrosis

Figure S2. Comparison of myofibroblast to macrophage cell count ratios in all fibrotic states, related to Figures 1 and 2

(A) Myofibroblast to macrophage neighborhood ratio (cell counts) calculated in all fibrotic conditions tested in Figures 1-2. These include myocardial infarction (n is as noted in Figure 1 legend), and transverse aortic constriction (n is as noted in Figure 2 legend). Boxes represent the quartiles, and the whiskers extend to include the minimum and maximum values, which central line represents the median value for each group. Individual dots represent biological replicates. Statistical analysis performed using the Mann-Whitney test with Bonferroni adjusted p-values when appropriate.

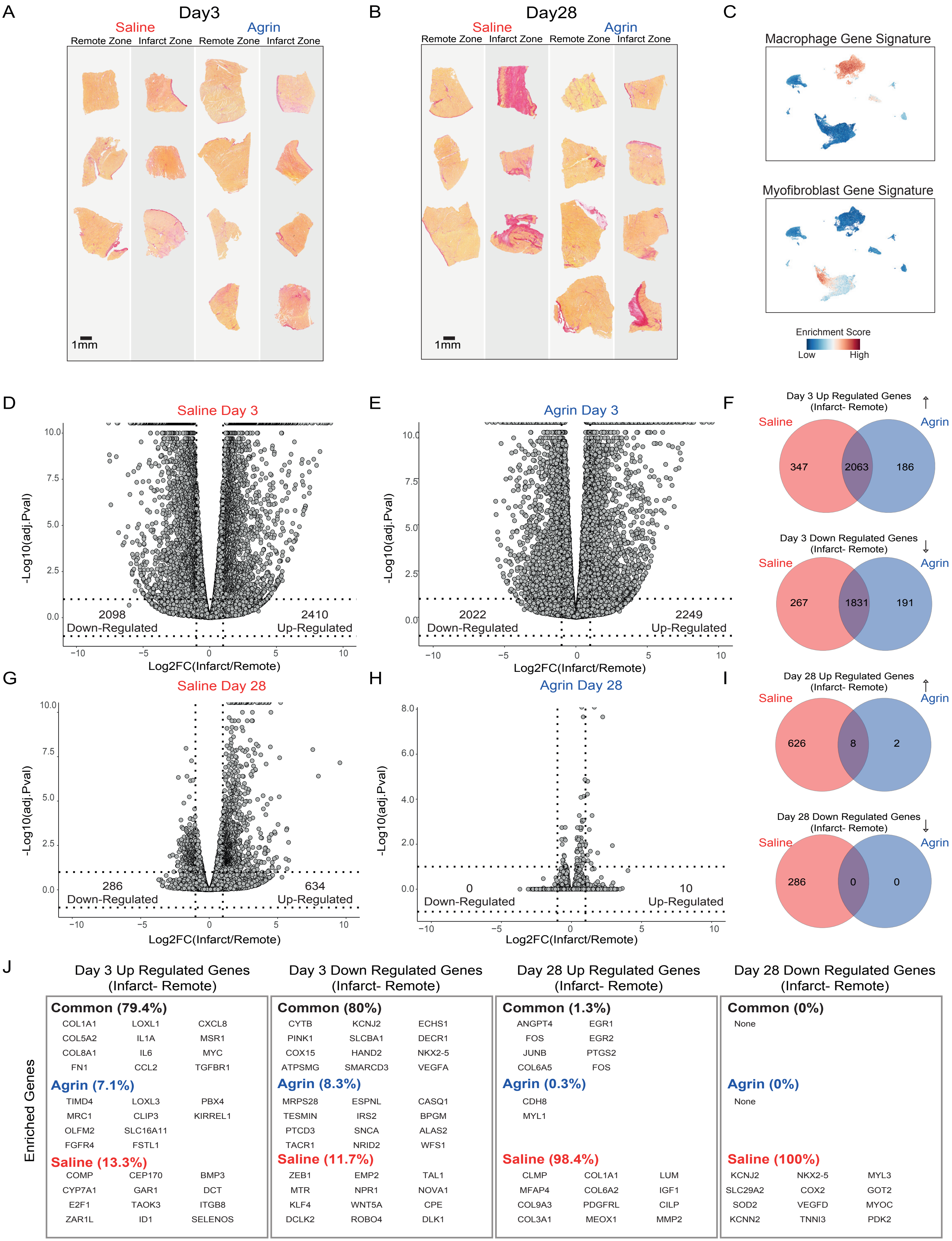
A
Day3
Saline
Agrin
Remote Zone
Infarct Zone
Remote Zone
Infarct Zone
1mm
B
Day28
Saline
Agrin
Remote Zone
Infarct Zone
Remote Zone
Infarct Zone
1mm
C
Macrophage Gene Signature
Myofibroblast Gene Signature
Enrichment Score
Low
High
D
Saline Day 3
-Log10(adj.Pval)
2098
Down-Regulated
2410
Up-Regulated
Log2FC(Infarct/Remote)
E
Agrin Day 3
-Log10(adj.Pval)
2022
Down-Regulated
2249
Up-Regulated
Log2FC(Infarct/Remote)
F
Day 3 Up Regulated Genes (Infarct- Remote)
Saline
Agrin
347
2063
186
Day 3 Down Regulated Genes (Infarct- Remote)
Saline
Agrin
267
1831
191
G
Saline Day 28
-Log10(adj.Pval)
286
Down-Regulated
634
Up-Regulated
Log2FC(Infarct/Remote)
H
Agrin Day 28
-Log10(adj.Pval)
0
Down-Regulated
10
Up-Regulated
Log2FC(Infarct/Remote)
I
Day 28 Up Regulated Genes (Infarct- Remote)
Saline
Agrin
626
8
2
Day 28 Down Regulated Genes (Infarct- Remote)
Saline
Agrin
286
0
0
J
Enriched Genes
Day 3 Up Regulated Genes (Infarct- Remote)
Common (79.4%)
COL1A1 LOXL1 CXCL8
COL5A2 IL1A MSR1
COL8A1 IL6 MYC
FN1 CCL2 TGFBR1
Agrin (7.1%)
TIMD4 LOXL3 PBX4
MRC1 CLIP3 KIRREL1
OLFM2 SLC16A11
FGFR4 FSTL1
Saline (13.3%)
COMP CEP170 BMP3
CYP7A1 GAR1 DCT
E2F1 TAOK3 ITGB8
ZAR1L ID1 SELENOS
Day 3 Down Regulated Genes (Infarct- Remote)
Common (80%)
CYTB KCNJ2 ECHS1
PINK1 SLCBA1 DECR1
COX15 HAND2 NKX2-5
ATPSMG SMARCD3 VEGFA
Agrin (8.3%)
MRPS28 ESPNL CASQ1
TESMIN IRS2 BPGM
PTCD3 SNCA ALAS2
TACR1 NRID2 WFS1
Saline (11.7%)
ZEB1 EMP2 TAL1
MTR NPR1 NOVA1
KLF4 WNT5A CPE
DCLK2 ROBO4 DLK1
Day 28 Up Regulated Genes (Infarct- Remote)
Common (1.3%)
ANGPT4 EGR1
FOS EGR2
JUNB PTGS2
COL6A5 FOS
Agrin (0.3%)
CDH8
MYL1
Saline (98.4%)
CLMP COL1A1 LUM
MFAP4 COL6A2 IGF1
COL9A3 PDGFRL CILP
COL3A1 MEOX1 MMP2
Day 28 Down Regulated Genes (Infarct- Remote)
Common (0%)
None
Agrin (0%)
None
Saline (100%)
KCNJ2 NKX2-5 MYL3
SLC29A2 COX2 GOT2
SOD2 VEGFD MYOC
KCNN2 TNNI3 PDK2

Figure S3. Differentially expressed genes in Saline or rhAgrin treated porcine hearts following-MI, related to Figure 3

(A-B) Representative sirius red staining images of remote and infarct zones, from all saline and rhAgrin treated hearts from day 3 (A) and 28 (B) post-MI used for fibrosis quantification (Figure 3D-E). Scale bars: 1 mm.

(C) Validation of cardiac macrophage and myofibroblast gene signatures (Table S5) used for deconvolution analysis in Figure 2I-J. Gene signature enrichment score was calculated for each gene set (cardiac macrophage and myofibroblast) and projected on a UMAP. Scores are presented on a linear scale from low to high and denoted by color (blue to red; linear scale).

(D-E) Volcano plots of differentially expressed genes (annotated genes that correspond with the following conditions: $|log2FoldChange| >= 1$, p-adjusted value < 0.05 and max raw counts > 30) between remote and infarct zones of day 3 Saline (D) and rhAgrin (E).

(F) Venn diagrams of common and unique up and down regulated genes between rhAgrin and Saline treated hearts from day 3 post-MI.

(G-H) Volcano plots of differentially expressed genes (annotated genes that correspond with the following conditions: $|log2FoldChange| >= 1$, p-adjusted value < 0.05 and max raw counts > 30) between remote and infarct zones of day 28 Saline (G) and rhAgrin (H).

(I) Venn diagrams of common and unique up and down regulated genes between rhAgrin and Saline treated hearts from day 28 post-MI.

(J) Highlighted genes for each Venn diagram analysis. % Are the number of genes presented in each category (common, agrin or saline) calculated from all differentially expressed genes in the corresponding Venn diagram. % Are the number of genes presented in each category (common,

rhAgrin or Saline) calculated from all differentially expressed genes in the corresponding Venn diagram.

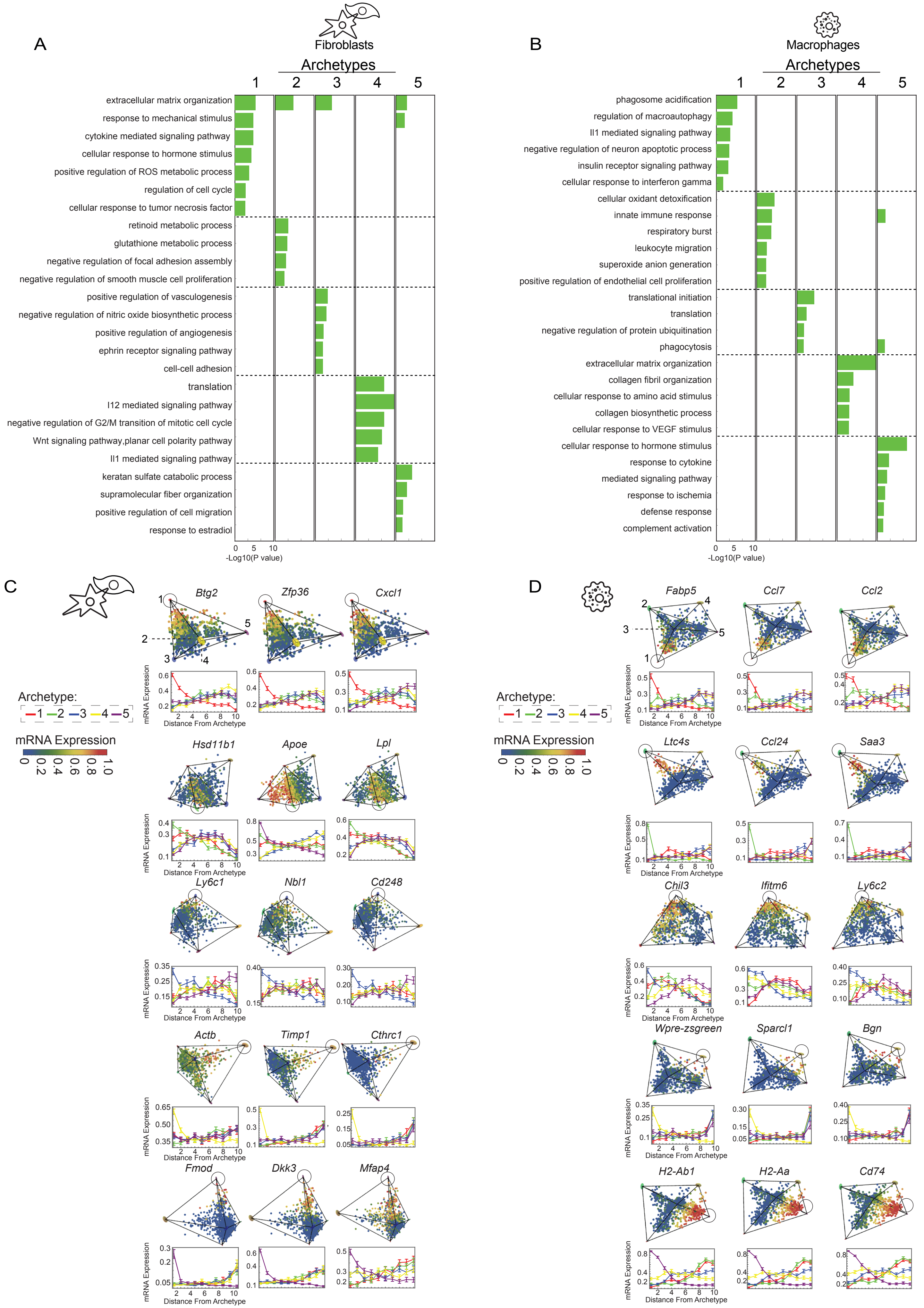

A
Fibroblasts
Archetypes
1 2 3 4 5
extracellular matrix organization
response to mechanical stimulus
cytokine mediated signaling pathway
cellular response to hormone stimulus
positive regulation of ROS metabolic process
regulation of cell cycle
cellular response to tumor necrosis factor
retinoid metabolic process
glutathione metabolic process
negative regulation of focal adhesion assembly
negative regulation of smooth muscle cell proliferation
positive regulation of vasculogenesis
negative regulation of nitric oxide biosynthetic process
positive regulation of angiogenesis
ephrin receptor signaling pathway
cell-cell adhesion
translation
I12 mediated signaling pathway
negative regulation of G2/M transition of mitotic cell cycle
Wnt signaling pathway,planar cell polarity pathway
Il1 mediated signaling pathway
keratan sulfate catabolic process
supramolecular fiber organization
positive regulation of cell migration
response to estradiol
0 5 10
-Log10(P value)
B
Macrophages
Archetypes
1 2 3 4 5
phagosome acidification
regulation of macroautophagy
Il1 mediated signaling pathway
negative regulation of neuron apoptotic process
insulin receptor signaling pathway
cellular response to interferon gamma
cellular oxidant detoxification
innate immune response
respiratory burst
leukocyte migration
superoxide anion generation
positive regulation of endothelial cell proliferation
translational initiation
translation
negative regulation of protein ubiquitination
phagocytosis
extracellular matrix organization
collagen fibril organization
cellular response to amino acid stimulus
collagen biosynthetic process
cellular response to VEGF stimulus
cellular response to hormone stimulus
response to cytokine
mediated signaling pathway
response to ischemia
defense response
complement activation
0 5 10
-Log10(P value)
C
Archetype:
1 2 3 4 5
mRNA Expression
0 0.2 0.4 0.6 0.8 1.0
Btg2 Zfp36 Cxcl1
Hsd11b1 Apoe Lpl
Ly6c1 Nbl1 Cd248
Actb Timp1 Cthrc1
Fmod Dkk3 Mfap4
mRNA Expression
Distance From Archetype
D
Archetype:
1 2 3 4 5
mRNA Expression
0 0.2 0.4 0.6 0.8 1.0
Fabp5 Ccl7 Ccl2
Ltc4s Ccl24 Saa3
Chil3 Ifitm6 Ly6c2
Wpre-zsgreen Sparcl1 Bgn
H2-Ab1 H2-Aa Cd74
mRNA Expression
Distance From Archetype

Figure S4. Days 0 and 3 post-MI archetype enrichment analysis, related to Figure 4

(A-B) Table of top enriched GO terms for the different fibroblast (A) and macrophage (B) archetypes and their p-values in log scale.

(C-D) Examples of top enriched genes per fibroblast (C) and (D) macrophage archetypes. The expression of each gene is plotted in two ways: upper panels- the cells are colored based on the expression levels within the tetrahedron where they are projected on the first three principal components; lower panels- expression is plotted across 10 bins as a function of the euclidean distance from each archetype in gene expression space. Red, green, blue, yellow and purple correspond to archetype numbers 1-5, respectively.

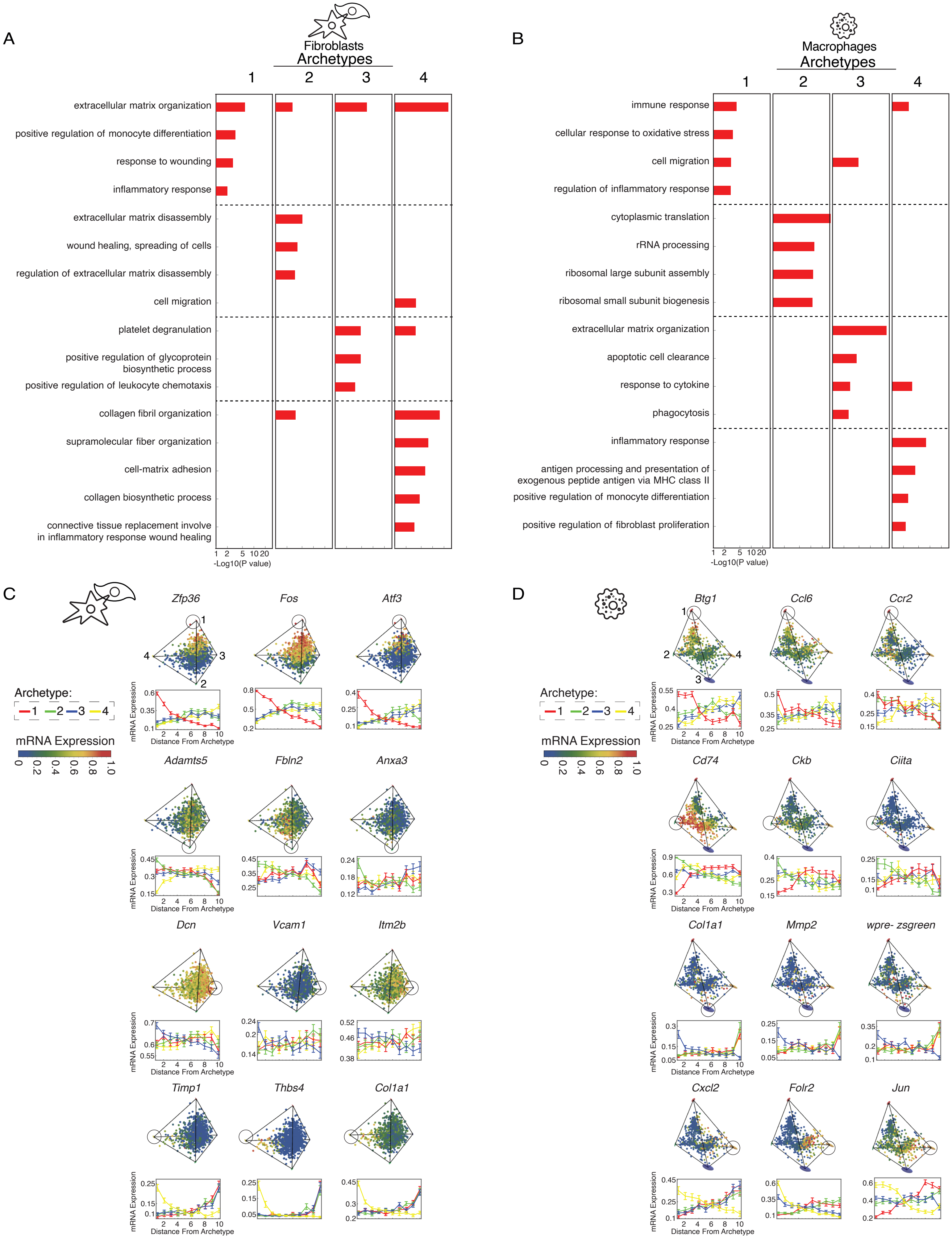
A
Fibroblasts
Archetypes
1
2
3
4
extracellular matrix organization
positive regulation of monocyte differentiation
response to wounding
inflammatory response
extracellular matrix disassembly
wound healing, spreading of cells
regulation of extracellular matrix disassembly
cell migration
platelet degranulation
positive regulation of glycoprotein biosynthetic process
positive regulation of leukocyte chemotaxis
collagen fibril organization
supramolecular fiber organization
cell-matrix adhesion
collagen biosynthetic process
connective tissue replacement involve in inflammatory response wound healing
1 2 5 10 20
-Log10(P value)
B
Macrophages
Archetypes
1
2
3
4
immune response
cellular response to oxidative stress
cell migration
regulation of inflammatory response
cytoplasmic translation
rRNA processing
ribosomal large subunit assembly
ribosomal small subunit biogenesis
extracellular matrix organization
apoptotic cell clearance
response to cytokine
phagocytosis
inflammatory response
antigen processing and presentation of exogenous peptide antigen via MHC class II
positive regulation of monocyte differentiation
positive regulation of fibroblast proliferation
1 2 5 10 20
-Log10(P value)
C
Archetype:
1 2 3 4
mRNA Expression
0 0.2 0.4 0.6 0.8 1.0
Zfp36
Fos
Atf3
Adamts5
Fbln2
Anxa3
Dcn
Vcam1
Itm2b
Timp1
Thbs4
Col1a1
mRNA Expression
Distance From Archetype
D
Archetype:
1 2 3 4
mRNA Expression
0 0.2 0.4 0.6 0.8 1.0
Btg1
Ccl6
Ccr2
Cd74
Ckb
Ciita
Col1a1
Mmp2
wpre- zsgreen
Cxcl2
Folr2
Jun
mRNA Expression
Distance From Archetype

Figure S5. Days 0 and 28 post-MI archetype enrichment analysis, related to Figure 4

(A-B) Table of top enriched GO terms for the different fibroblast (A) and macrophage (B) archetypes and their P-values in log scale.

(C-D) Examples of top enriched genes per fibroblast (C) and (D) macrophage archetypes. The expression of each gene is plotted in two ways: upper panels- the cells are colored based on the expression levels within the tetrahedron where they are projected on the first three principal components; lower panels- expression is plotted across 10 bins as a function of the euclidean distance from each archetype in gene expression space. Red, green, blue, and yellow correspond to archetype numbers 1-4, respectively.

Figure S5- supplementary text: We note that archetype 3 in macrophages (Figure S5D), associated with phagocytotic functions, shows expression of prominent genes usually expressed in fibroblasts (*Col1a1*). It also shows expression of an epicardial derived cells lineage tracer (wpre-zsgreen) introduced in the experiment of *Forte et al.* These authors indeed note the existence of a myeloid-myofibroblast cluster in their scRNAseq analysis [46].

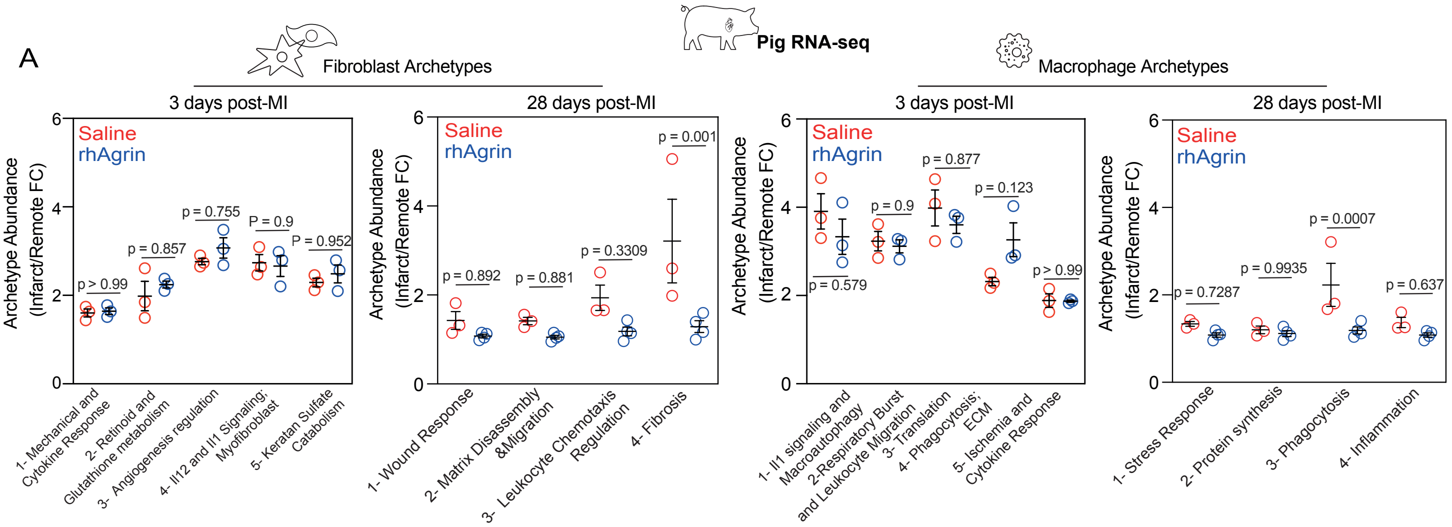
A
Pig RNA-seq
Fibroblast Archetypes
Macrophage Archetypes
3 days post-MI
28 days post-MI
Saline
rhAgrin
Archetype Abundance
(Infarct/Remote FC)
p > 0.99
p = 0.857
p = 0.755
P = 0.9
P = 0.952
1- Mechanical and Cytokine Response
2- Retinoid and Glutathione metabolism
3- Angiogenesis regulation
4- Il12 and Il1 Signaling; Myofibroblast
5- Keratan Sulfate Catabolism
p = 0.892
p = 0.881
p = 0.3309
p = 0.001
1- Wound Response
2- Matrix Disassembly &Migration
3- Leukocyte Chemotaxis Regulation
4- Fibrosis
p = 0.579
p = 0.9
p = 0.877
p = 0.123
p > 0.99
1- Il1 signaling and Macroautophagy
2-Respiratory Burst and Leukocyte Migration
3- Translation
4- Phagocytosis, ECM
5- Ischemia and Cytokine Response
p = 0.7287
p = 0.9935
p = 0.0007
p = 0.637
1- Stress Response
2- Protein synthesis
3- Phagocytosis
4- Inflammation

Figure S6. Assessment of pareto archetypes in Pig mRNA-seq, related to Figure 4

(A) Deconvolution of Bulk mRNA sequencing of pig hearts following MI of either rhAgrin (blue) or Saline treated (red) samples. Macrophage and fibroblast archetypes (from both days: 0+ 3 and 0+28 analysis, Figure 4) abundance were assessed by gene signatures (Table S6), between infarct and remote zones (STAR Methods). Statistical analysis performed using two-way ANOVA with Sidak's adjusted p-values.

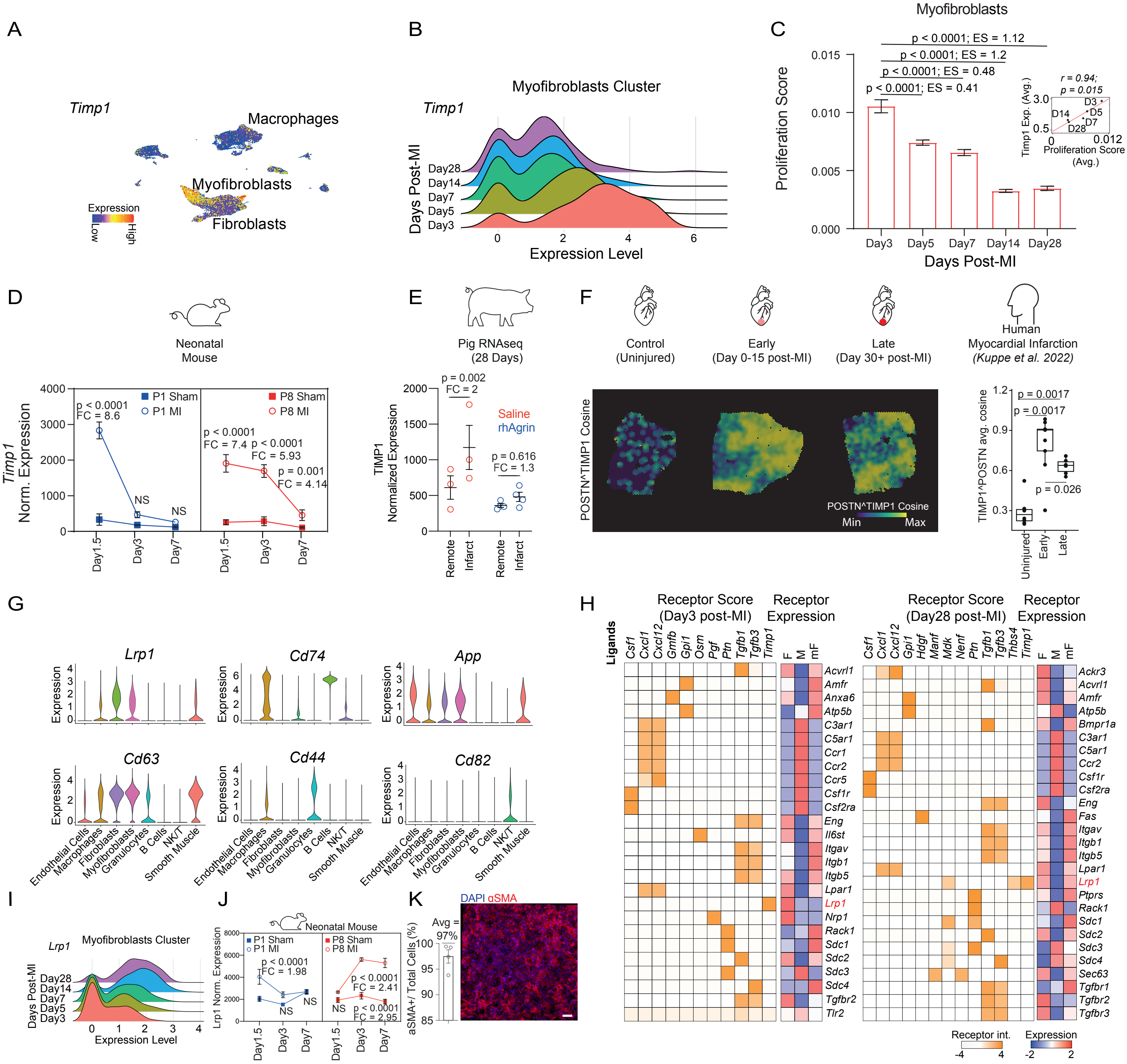
A
Timp1
Macrophages
Myofibroblasts
Fibroblasts
Expression
Low
High
B
Myofibroblasts Cluster
Timp1
Days Post-MI
Day28
Day14
Day7
Day5
Day3
Expression Level
C
Myofibroblasts
p < 0.0001; ES = 1.12
p < 0.0001; ES = 1.2
p < 0.0001; ES = 0.48
p < 0.0001; ES = 0.41
Proliferation Score
Day3
Day5
Day7
Day14
Day28
Days Post-MI
r = 0.94;
p = 0.015
Timp1 Exp. (Avg.)
Proliferation Score (Avg.)
D
Neonatal Mouse
Timp1 Norm. Expression
P1 Sham
P1 MI
P8 Sham
P8 MI
p < 0.0001
FC = 8.6
NS
NS
p < 0.0001
FC = 7.4
p < 0.0001
FC = 5.93
p = 0.001
FC = 4.14
Day1.5
Day3
Day7
E
Pig RNAseq (28 Days)
TIMP1 Normalized Expression
p = 0.002
FC = 2
Saline
rhAgrin
p = 0.616
FC = 1.3
Remote
Infarct
F
Control (Uninjured)
Early (Day 0-15 post-MI)
Late (Day 30+ post-MI)
Human Myocardial Infarction
(Kuppe et al. 2022)
POSTN^TIMP1 Cosine
Min
Max
TIMP1^POSTN avg. cosine
p = 0.0017
p = 0.0017
p = 0.026
Uninjured
Early
Late
G
Lrp1
Cd74
App
Cd63
Cd44
Cd82
Expression
Endothelial Cells
Macrophages
Fibroblasts
Myofibroblasts
Granulocytes
B Cells
NK/T
Smooth Muscle
H
Ligands
Receptor Score (Day3 post-MI)
Receptor Expression
Receptor Score (Day28 post-MI)
Csf1 Cxcl1 Cxcl12 Gmfb Gpi1 Osm Pgf Ptn Tgfb1 Tgfb3 Timp1
F M mF
Acvrl1 Amfr Anxa6 Atp5b C3ar1 C5ar1 Ccr1 Ccr2 Ccr5 Csf1r Csf2ra Eng Il6st Itgav Itgb1 Itgb5 Lpar1 Lrp1 Nrp1 Rack1 Sdc1 Sdc2 Sdc3 Sdc4 Tgfbr2 Tlr2
Csf1 Cxcl1 Cxcl12 Gpi1 Hdgf Manf Mdk Nenf Ptn Tgfb1 Tgfb3 Thbs4 Timp1
Ackr3 Acvrl1 Amfr Atp5b Bmpr1a C3ar1 C5ar1 Ccr2 Csf1r Csf2ra Eng Fas Itgav Itgb1 Itgb5 Lpar1 Lrp1 Ptprs Rack1 Sdc1 Sdc2 Sdc3 Sdc4 Sec63 Tgfbr1 Tgfbr2 Tgfbr3
Receptor int.
Expression
I
Lrp1
Myofibroblasts Cluster
Days Post-MI
Expression Level
J
Neonatal Mouse
Lrp1 Norm. Expression
P1 Sham
P1 MI
P8 Sham
P8 MI
p < 0.0001
FC = 1.98
NS
NS
NS
p < 0.0001
FC = 2.41
p < 0.0001
FC = 2.95
K
DAPI αSMA
Avg = 97%
aSMA+/ Total Cells (%)

Figure S7. Timp1 expression dynamics following MI in mice pigs and humans, related to Figure 5

(A) Single-cell mRNA expression of *Timp1* by cardiac interstitial cells shows dominant expression in cardiac myofibroblasts. Scale denotes normalized mRNA expression. Dataset used is described in Figure 1B and Figure S1A-B.

(B) *Timp1* mRNA expression dynamics after MI quantified between day 3 and day 28 in cardiac myofibroblasts cluster.

(C) The dynamic of cardiac myofibroblasts proliferation was assessed using the scRNAseq data described in Figure 1B and Figure S1A-B (STAR Methods). Small right panel- We calculated the Pearson correlation coefficient (r) between the average myofibroblasts proliferation score per day, and the average *Timp1* expression presented in Figure S7B. Statistical analysis performed using either the Mann-Whitney test, with p-values were adjusted for multiple testing using the Benjamini and Hochberg procedure or Pearson correlation test (small right panel). ES- effect size.

(D) *Timp1* mRNA expression in the publicly available bulk mRNA-sequencing [112]. P1 (blue) and P8 (red) mice underwent Sham (squares) or MI (circles) injury by LAD ligation and ventricles were sampled on days 1.5, 3 and 7 post-MI for sequencing. Data is shown as normalized mRNA expression. (n = 3 per time-point, per age/ treatment). Statistical analysis was performed using DESeq2, p-values were adjusted for multiple testing using the Benjamini and Hochberg procedure (STAR Methods). FC- fold change.

(E) Pig TIMP1 normalized mRNA expression at day 28 post-MI, in infarct and remote zones of rhAgrin (blue) and Saline (red) treated hearts. Circles denote biological replicates. FC = fold-change. Statistical analysis was performed using DESeq2, p-values were adjusted for multiple testing using the Benjamini and Hochberg procedure (STAR Methods).

(F) Spatial colocalization of the TIMP1-POSTN gene pair was calculated in human left ventricle spatial transcriptomics slides (Visium) of patients following acute-MI and non-transplanted donor hearts. Samples were divided based on time following MI as either: uninjured (n = 10), Early (days 0-15 post-MI; n = 6) and Late (30 days+; n = 6). Gene pair was quantified using spatially-weighted cosine similarity score using LIANA+[76] (STAR Methods). Statistical analysis used the Wilcoxon test with Benjamini-Hochberg adjusted p-values.

representative Human left ventricle spatial transcriptomics slides (Visium) of patients following acute-myocardial infarction (MI) and non-transplanted donor heart. Samples were divided based on time following MI as either: uninjured (n = 10), Early (days 0-15 post-MI; n = 6) and Late (30 days+; n = 6). (B) Abundance of fibroblasts and myeloid cells was quantified based on deconvolution scores of cell-types per spot (STAR Methods). Myofibroblast were calculated as enrichment of the mean myofibroblast state score within spots with a minimal 10% value of cell-type abundance (STAR Methods).

Spatial colocalization of the TIMP1-POSTN gene pair was quantified using spatially weighted cosine similarity via the spatial_neighbors function in liana-py v1.1.0, with bandwidth and expression cut-offs set to 150 and 0.1, respectively.

(G) Single-cell mRNA expression of TIMP1 receptors: *Lrp1, Cd63, Cd82, Cd74, App* and *Cd44* by cardiac interstitial cells shows dominant expression of *Cd63*, *App* and *Lrp1* in cardiac myofibroblasts.

(H) Heatmap of potential receptors based on cardiac fibroblast (F), macrophage (M) and myofibroblast (mF) on days 3 (left) and 28 (right) post-MI. These data are complementary to Figure 5D. Receptor interaction score and average receptor expression per cell type are presented as mean± SD (by either white-orange scale or blue-red scale, respectively)

(I) Single-cell mRNA expression of *Lrp1* by cardiac interstitial cells shows dominant expression in cardiac myofibroblasts. Scale denotes normalized mRNA expression. Dataset used is described in Figure 1B and Figure S1A-B.

(J) *Lrp1* mRNA expression in the publicly available bulk mRNA-sequencing [112]. P1 (blue) and P8 (red) mice underwent Sham (squares) or MI (circles) injury by LAD ligation and ventricles were sampled on days 1.5, 3 and 7 post-MI for sequencing. Data is shown as normalized mRNA expression. (n = 3 per time-point, per age/ treatment). Statistical analysis was performed using DESeq2, p-values were adjusted for multiple testing using the Benjamini and Hochberg procedure (STAR Methods). FC- fold change.

(K) Representative images of primary cardiac myofibroblasts cultures (day 4 of culture) related to Figure 5I. Bar plot presents the fraction of myofibroblasts (aSMA+, red) of total nuclei (DAPI, blue) identified (%) at day 4 (n = 4 replicates, mean cells=4253±749 SEM). Scale bar = 200μm.

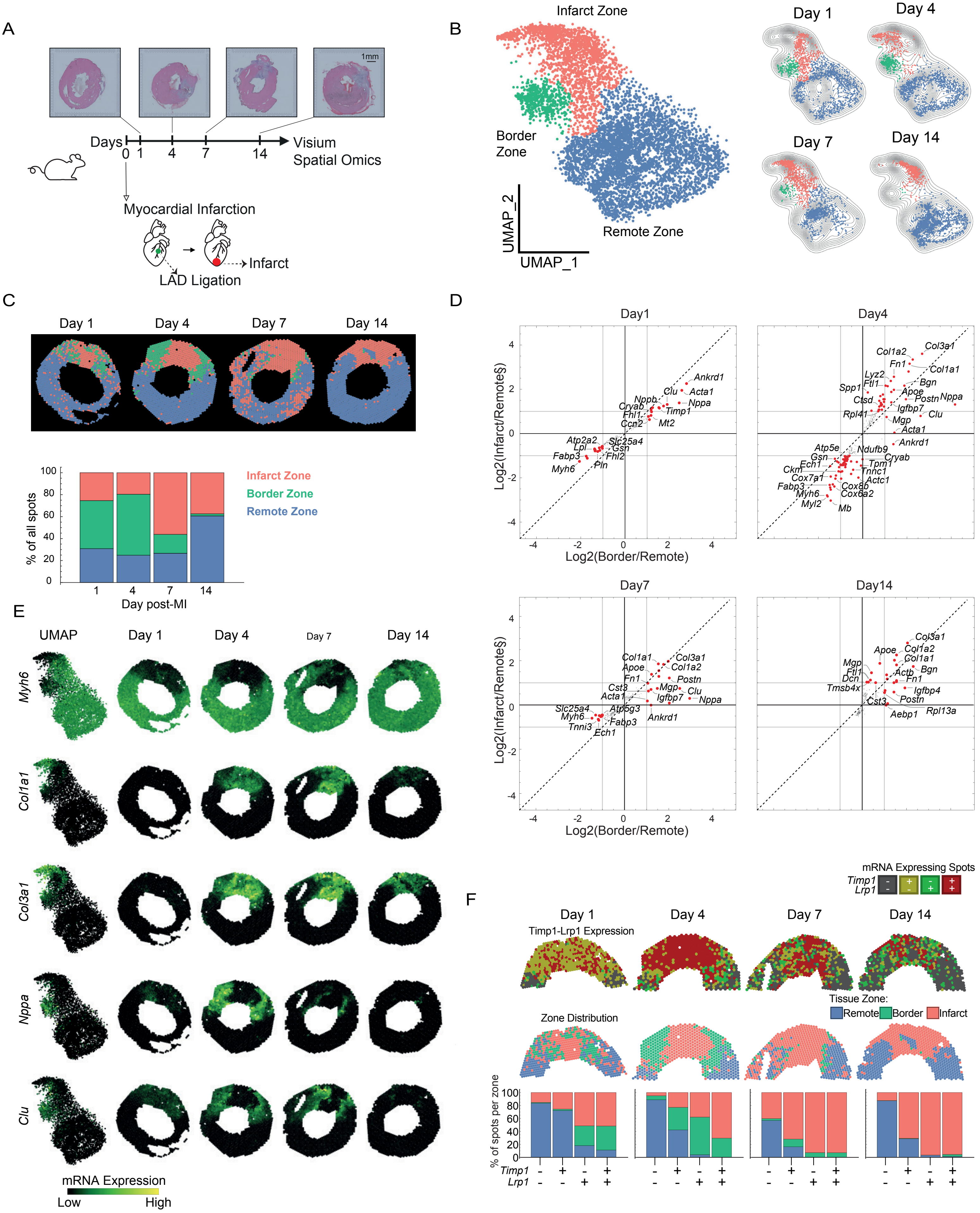
A
1mm
Days 0 1 4 7 14
Visium Spatial Omics
Myocardial Infarction
LAD Ligation
Infarct
B
Infarct Zone
Border Zone
Remote Zone
UMAP_1
UMAP_2
Day 1
Day 4
Day 7
Day 14
C
Day 1 Day 4 Day 7 Day 14
% of all spots
Day post-MI
Infarct Zone
Border Zone
Remote Zone
D
Day1
Day4
Day7
Day14
Log2(Infarct/Remote$)
Log2(Border/Remote)
E
UMAP Day 1 Day 4 Day 7 Day 14
Myh6
Col1a1
Col3a1
Nppa
Clu
mRNA Expression
Low High
F
mRNA Expressing Spots
Timp1
Lrp1
Day 1 Day 4 Day 7 Day 14
Timp1-Lrp1 Expression
Zone Distribution
Tissue Zone:
Remote Border Infarct
% of spots per zone

Figure S8. Visium spatial transcriptomics reveals *Timp1-Lrp1* co-expression persists in the infarct zone over time, related to Figure 5

(A) Experimental design of adult ICR mice hearts who underwent myocardial infarction (MI) and processed by Visium spatial transcriptomics analysis at 4 different time points following injury [at days: 1 (n = 1), 4 (n = 1), 7 (n = 1) and 14 (n = 1)]; Top panel: representative Hematoxylin and Eosin (H&E) stained section. Scale bar, 1 mm.

(B) Left panel- uniform manifold approximation and projection (UMAP) plot of total spots from all timepoints combined day1 (n = 1516), day 4 (n = 1596), day 7 (n = 1510), day 14 (n = 2117) . Spots were annotated as 3 distinct gene-expression clusters [Infarct Zone (orange), Border Zone (green), Remote (Healthy) Zone (blue)]. Right panel- UMAP plots of the 3 clusters according to time following MI with density contour based on smooth kernel distribution.

(C) Top- Clusters (Infarct, border and remote zones) represented by their spatial coordinates per timepoint. Bottom- Cluster distribution (% of total spots per timepoint).

(D) Spatio-temporal differential gene expression analysis of adult mice following-MI. Log2 fold-change (FC) was calculated per area in comparison to the corresponding remote zone per section (used as internal control) (Infarct/Remote, Border/Remote). Statistical analysis performed using the Mann-Whitney test with Bonferroni adjusted p-values. Differentially expressed genes (red spots) were defined as log2FC > 1 and significant adjusted p-values (<0.05). Small black dots represent non-differentially expressed genes.

(E) Visium spatial cluster defining genes (Remote zone- *Myh6*, Infarct zone- *Col1a1*, *Col3a1*; Border zone- *Nppa*, *Clu*) projected on all sections and combined UMAP. mRNA expression is denoted by color (black to yellow). Each slice was rotated so that the infarct zone median (defined by infarct zone spots) is positioned upwards.

(F) Top panels- *Timp1* and *Lrp1* spatio-temporal distribution after MI. Positive spots (either *Timp1*, *Lrp1* or both) were defined as spots with expression, of each gene, higher than the median expression over all slices. Middle panels- zone distribution. Bottom panels- quantification of *Timp1*-*Lrp1* spot distribution, divided per zone. % of spots (based on *Timp1*-*Lrp1* expression) per zone are presented in a percentile graph.

For all spatial spot distribution plots (panels: C, E and F) each slice was rotated so that the infarct zone median (defined by infarct zone spots) is positioned upwards.

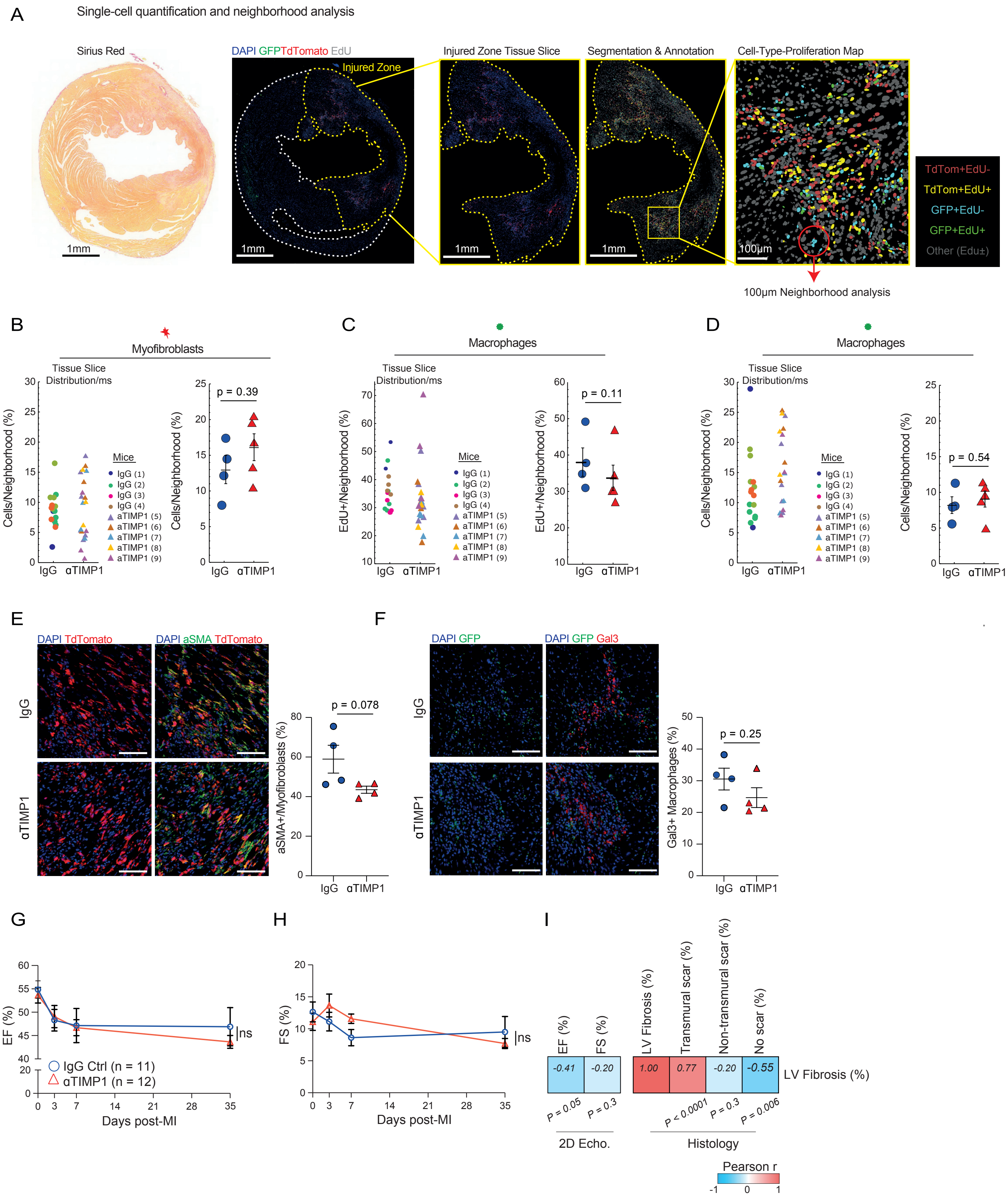
A
Single-cell quantification and neighborhood analysis
Sirius Red
DAPI GFP TdTomato EdU
Injured Zone
Injured Zone Tissue Slice
Segmentation & Annotation
Cell-Type-Proliferation Map
1mm
100µm
TdTom+EdU-
TdTom+EdU+
GFP+EdU-
GFP+EdU+
Other (Edu±)
100µm Neighborhood analysis
B
Myofibroblasts
Tissue Slice Distribution/ms
Cells/Neighborhood (%)
p = 0.39
Mice
IgG (1)
IgG (2)
IgG (3)
IgG (4)
aTIMP1 (5)
aTIMP1 (6)
aTIMP1 (7)
aTIMP1 (8)
aTIMP1 (9)
IgG
αTIMP1
C
Macrophages
EdU+/Neighborhood (%)
p = 0.11
D
Macrophages
p = 0.54
E
DAPI TdTomato
DAPI aSMA TdTomato
aSMA+/Myofibroblasts (%)
p = 0.078
F
DAPI GFP
DAPI GFP Gal3
Gal3+ Macrophages (%)
p = 0.25
G
EF (%)
IgG Ctrl (n = 11)
αTIMP1 (n = 12)
Days post-MI
ns
H
FS (%)
Days post-MI
ns
I
EF (%)
FS (%)
LV Fibrosis (%)
Transmural scar (%)
Non-transmural scar (%)
No scar (%)
-0.41
-0.20
1.00
0.77
-0.20
-0.55
LV Fibrosis (%)
P = 0.05
P = 0.3
P < 0.0001
P = 0.3
P = 0.006
2D Echo.
Histology
Pearson r
-1
0
1

Figure S9. αTIMP1 treatment did not attenuate macrophage abundance, proliferation, state, or myofibroblast activation, related to Figure 5

(A) Neighborhood analysis pipeline for double-reporter mice (MAMY) following MI and IgG control/ αTIMP1 treatment (related to Figure 5J-L, Figure S9B-D). from left to right- annotation of immunofluorescence of infarct zone tissue slices were based on consecutive histological sections stained with sirius red. 2-6 tissue slices were analyzed for each biological replicate. Cells were segmented (based on nuclear DAPI stain, blue), annotated for cell type and proliferation state (STAR Methods) and a cell-type-proliferation map was generated to represent the macrophage, myofibroblast and other cell-type, and their proliferative state in the correct spatial localization: proliferative macrophages (GFP+EdU+, green), non-proliferative macrophage (GFP+EdU+, light-blue), proliferative myofibroblasts (TdTomato+EdU+, yellow), non-proliferative myofibroblasts (TdTomato+EdU-, red) and others (GFP-TdTomato-EdU±, grey). For each cell identified the cell composition within its neighborhood: measured as a 50µm radius (100µm in diameter) from the center of each cell was determined. Percentages of the total abundance of each cell type and their proliferative states were determined.

(B) Quantification of myofibroblasts (total TdTomato+) abundance (% of cells) per cellular neighborhood (100µm in diameter) in the infarct zone of αTIMP1 (n = 5) or IgG control (n = 4) treated MAMY hearts (STAR Methods). Left panel- distribution of data per tissue slice per biological replicate. Each color represents a biological replicate. Right panel- average TdTomato+EdU±/neighborhood per biological replicate. Results are represented as mean± SEM. Blue circles- IgG control; red triangle- αTIMP1. Statistical analysis performed using the Mann-Whitney test.

(C) Quantification of proliferative macrophage (GFP+EdU+) abundance (% of cells) per cellular neighborhood (100µm in diameter) in the infarct zone of αTIMP1 (n = 5) or IgG control (n = 4) treated MAMY hearts (STAR Methods). Left panel- distribution of data per tissue slice per biological replicate. Each color represents a biological replicate. Right panel- average GFP+EdU+/neighborhood per biological replicate. Results are represented as mean± SEM. Blue circles- IgG control; red triangle- αTIMP1. Statistical analysis performed using the Mann-Whitney test.

(D) Quantification of macrophage (total GFP+) abundance (% of cells) per cellular neighborhood (100µm in diameter) in the infarct zone of αTIMP1 (n = 5) or IgG control (n = 4) treated MAMY hearts (STAR Methods). Left panel- distribution of data per tissue slice per biological replicate. Each color represents a biological replicate. Right panel- average GFP+EdU±/neighborhood per biological replicate. Results are represented as mean± SEM. Blue circles- IgG control; red triangle- αTIMP1. Statistical analysis performed using the Mann-Whitney test.

(E) Quantification of alpha smooth actin (aSMA) expressing myofibroblasts (TdTomato+ aSMA+) abundance (% of cells) in the infarct zone of αTIMP1 (n = 4; 1244±251.8 SEM cells per replicate) or IgG control (n = 4; 1070±176.3 SEM TdTomato+ cells per replicate) treated MAMY hearts. Data are represented as the total sum of aSMA+TdTomato+ cells out of total TdTomato+ cells sampled in each biological replicate. Statistical analysis performed using two-tailed unpaired t-test.

(F) Quantification of Galectin-3 (Gal3) expressing macrophage (GFP+Gal3+) abundance (% of cells) in the infarct zone of αTIMP1 (n = 4; 943.8±191.5 SEM GFP+ cells per replicate) or IgG control (n = 4; 1088±333.2 SEM GFP+ cells per replicate) treated MAMY hearts. Data are

represented as the total sum of GFP+Gal3+ cells out of total GFP+ cells sampled in each biological replicate. Statistical analysis performed using two-tailed unpaired t-test.

(G-H) Temporal echocardiography (echo) systolic measurements of (G) Ejection fraction (EF; %) and (H) fractional shortening (FS; %) of the experiment presented in Figure 5M. Analysis was performed on MI operated adult mice (αTIMP1, n = 12; IgG, n = 11) (Methods). Echo function was measured repeatedly on baseline, and days 3, 7 and 35 post-MI. Red- αTIMP1; Blue- IgG. Results are represented as mean± SEM. Statistical analysis performed using two-way ANOVA with Sidak's adjusted p-values.

(I) Correlation matrix between left ventricle (LV) fibrosis measurements (in %; presented in Figure 5O) to either fibrotic parameters (scar-class analysis; presented in Figure 5O) or systolic 2D Echo data (EF, FS; presented in Figure S9G-H). Scale bar (blue to red), represent negative (-1) to positive (1) Pearson correlation coefficient values. Both Pearson correlation coefficient and p-values are indicated for each correlation pair.

Note S1: Specificity, label efficacy, and off-target effects of the mouse lines comprising the MAMY mouse.

The myofibroblast lineage reporter ($Postn^{MCM/+}Rosa26^{TdTomto/+}$): The $Postn^{MCM}$ mouse line was established in 2016 by *Kanisicak, et al.*[1], and has been used by multiple labs as a lineage reporter and as a tool to specifically knock-out genes in cardiac myofibroblasts. It has been cited >800 times.

Kanisicak, et al. showed that Periostin is exclusively expressed in injured areas and is a specific marker of myofibroblasts in the heart and other tissues following injury. Expression of eGFP under the control of the $Postn^{MCM}$ cassette and exposure of these mice to tamoxifen did not label almost any cells in the uninjured heart. In other tissues, minimal off-target labeling was further demonstrated in the absence of injury. This includes skeletal muscle, kidney, lung, liver, and skin following 8 weeks of tamoxifen exposure. The $Postn^{MCM}$ cassette successfully labeled cells only at the infarcted area (7 days post-MI). Whole mount tracking of these hearts up to 7 days following MI, revealed the progressive accumulation of eGFP+ cells in the infarct zone over time. These data were reproduced by a study that tested the dynamics of *Postn* gene expression following MI in mice[2].

Kanisicak, et al. further characterized the label efficiency and specificity of the $Postn^{MCM}$ lineage tracing system in the context of acute, (namely, myocardial infarction; MI) and chronic (namely, transverse aortic constriction; TAC or AngII osmotic pumps) injuries.

In the context of MI, using histological sections stained by immunofluorescence, *Kanisicak, et al.* show that 2 weeks following MI induction and tamoxifen administration, most of the eGFP-labeled cells were Vimentin (fibroblast marker) positive (±98%), >50% were PDGFRa (fibroblast marker)

positive and ±80% were aSMA (activated fibroblast marker) positive. But none were CD31 (endothelial cells marker), or CD45 (immune cells marker). Consistent with these results the authors further bolster their findings with flow cytometry analysis.

To obtain higher granularity in the eGFP+ cells characterization, *Kanisicak, et al.* sorted 3 populations of cells and analyzed them by RNA-sequencing: 1) Resident fibroblasts defined by CD31-CD45- markers. 2) Activated injured zone cells, defined by Postn-CD31-CD45- markers, and 3) Activated *Postn*-tracked eGFP+ cells that were also CD31-CD45-. Indeed among these cells, only the postn-traced eGFP+ cells significantly expressed myofibroblast-specific genes such as *aSMA*, *Lox,* and *Fibronectin* [3,4].

In the context of chronic cardiac injury (TAC) the authors were able to show that eGFP+ cells were Vimentin and aSMA positive, But not CD31 or CD45 positive. These data were consistent with the demonstrated specificity of the lineage reporter following MI.

In summary, the Postn^{MCM} lineage tracing system is a robust and validated tool for specifically labeling myofibroblasts in the context of cardiac injury and other tissue injuries. The work by *Kanisicak, et al.*, supported by subsequent studies[5–14], demonstrates that Periostin expression is specific to myofibroblasts in injured tissues, with minimal off-target effects in uninjured regions or non-myofibroblast cells. This system has been demonstrated to reliably identify myofibroblasts across multiple experimental models, including myocardial infarction, pressure overload, and fibrotic responses in other organs[5–14].

The monocyte/macrophage reporter (Cx3cr1$^{GFP/+}$): The Cx3cr1^{GFP} mouse model is widely used in the field of monocyte/macrophage biology. It was established by *Jung, et al.*[15] , a paper cited >2800 times. In the context of the heart, the Cx3cr1^{GFP} mouse line has been extensively validated

to specifically and reliably label all cardiac macrophage subsets through the expression of green fluorescent protein (GFP)[16–20].

In 2012, *Pinto, et al.*[19] established the specificity of Cx3cr1^{GFP+} cells in cardiac tissue, as ±95% of these cells also express myeloid cell markers, such as CD45 and CD11b. They further show that most of these cells were express F4/80, MHCII, CD14, and CD86- highly abundant myeloid and macrophage markers. Importantly, these cells did not express NK1.1, B220, or CD3e- NK, B, and T cell markers.

In 2014, *Malawi, et al.*[20] repeated these results and established that >80% of cardiac macrophages, defined by core macrophage markers (CD64, F4/80, and MerTK) are indeed GFP+ cells.

In 2017, *Hulsmans, et al.* [18], repeated these results using a flow cytometry approach, establishing that CD45+CD31-CD11b+Lin-Ly6G-F4/80+ cells were indeed GFP+. importantly, CD45-CD31-MEFSK4+ cells (fibroblasts) were negative for GFP.

Hulsmans, et al., then sorted (by flow cytometry) either GFP+ cells, total macrophages, or fibroblasts and performed real-time qPCR for key macrophage, fibroblast, and myofibroblast genes. They found that GFP+ cells were expressing macrophage genes (i.e. *Csf1r, F4/80, LysM, MerTK, Cd64,* etc.) as the sorted macrophages, and did not express fibroblast (i.e. *Col1a2, Col3a1, Ddr2*) or myofibroblast genes (i.e. *Postn*).

In 2018, *Walter, et al.* [16], sorted GFP+ cells from cryo-injured (a form of acute cardiac injury) Cx3cr1^{GFP} hearts and performed RNA-sequencing at different time points following injury. This experiment allowed them to observe in detail the gene expression of GFP+ cells in the steady-state and injured heart. Indeed, the cardiac GFP+ cells gene expression strongly correlated with sorted

macrophage RNA-sequencing data from a variety of tissues including the lung, liver, spleen, and peritoneum.

In summary, the MAMY mouse model combines two validated and widely adopted genetic tools: the Cx3cr1^{GFP} system, which has been used in thousands of studies to reliably and specifically label macrophages, including cardiac macrophages,[16,18–20] and the Postn^{MCM} lineage reporter system, which is highly specific for identifying myofibroblasts in cardiac and other injured tissues[1,5–14]. Demonstrated through immunophenotyping, flow cytometry, and transcriptomic analyses in multiple studies, The Cx3cr1^{GFP} reporter has consistently shown robust specificity for monocytes/macrophages, with minimal off-target labeling of non-myeloid cell types including endothelial cells, lymphocytes, fibroblasts, and other stromal cells. Similarly, the Postn^{MCM} system has been shown to exclusively label myofibroblasts in the context of cardiac injury, with negligible labeling of other cell types. Together, the established specificity of these models ensures that the data generated using this model accurately captures cardiac myofibroblasts and all monocytes/macrophages in the injured zones, and further supports the validity of our findings using the MAMY mouse line.

References

1. Kanisicak, O., Khalil, H., Ivey, M.J., Karch, J., Maliken, B.D., Correll, R.N., Brody, M.J., Lin, S.-C.J., Aronow, B.J., Tallquist, M.D., et al. (2016). Genetic lineage tracing defines myofibroblast origin and function in the injured heart. Nat. Commun. *7*, *12*260. https://doi.org/10.1038/ncomms12260.
2. Gil, H., Goldshtein, M., Etzion, S., Elyagon, S., Hadad, U., Etzion, Y., and Cohen, S. (2022). Defining the timeline of periostin upregulation in cardiac fibrosis following acute myocardial infarction in mice. Sci. Rep. 12, 21863. https://doi.org/10.1038/s41598-022-26035-y.
3. Forte, E., Skelly, D.A., Chen, M., Daigle, S., Morelli, K.A., Hon, O., Philip, V.M., Costa, M.W., Rosenthal, N.A., and Furtado, M.B. (2020). Dynamic Interstitial Cell Response during Myocardial Infarction Predicts Resilience to Rupture in Genetically Diverse Mice. Cell Reports *30*, 3149-3163.e6. https://doi.org/10.1016/j.celrep.2020.02.008.
4. Farbehi, N., Patrick, R., Dorison, A., Xaymardan, M., Janbandhu, V., Wystub-Lis, K., Ho, J.W., Nordon, R.E., and Harvey, R.P. (2019). Single-cell expression profiling reveals dynamic flux of cardiac stromal, vascular and immune cells in health and injury. eLife 8, e43882. https://doi.org/10.7554/elife.43882.
5. Khalil, H., Kanisicak, O., Prasad, V., Correll, R.N., Fu, X., Schips, T., Vagnozzi, R.J., Liu, R., Huynh, T., Lee, S.-J., et al. (2017). Fibroblast-specific TGF-β–Smad2/3 signaling underlies cardiac fibrosis. J. Clin. Investig. *127*, 3770–3783. https://doi.org/10.*117*2/jci94753.
6. Fu, X., Khalil, H., Kanisicak, O., Boyer, J.G., Vagnozzi, R.J., Maliken, B.D., Sargent, M.A., Prasad, V., Valiente-Alandi, I., Blaxall, B.C., et al. (2018). Specialized fibroblast differentiated states underlie scar formation in the infarcted mouse heart. J Clin Invest *128*, 2127–2143. https://doi.org/10.1172/jci98215.
7. Aghajanian, H., Kimura, T., Rurik, J.G., Hancock, A.S., Leibowitz, M.S., Li, L., Scholler, J., Monslow, J., Lo, A., Han, W., et al. (2019). Targeting Cardiac Fibrosis with Engineered T cells. Nature *573*, 430–433. https://doi.org/10.1038/s41586-019-1546-z.
8. Meng, Q., Bhandary, B., Bhuiyan, M.S., James, J., Osinska, H., Valiente-Alandi, I., Shay-Winkler, K., Gulick, J., Molkentin, J.D., Blaxall, B.C., et al. (2018). Myofibroblast-Specific TGFβ Receptor II Signaling in the Fibrotic Response to Cardiac Myosin Binding Protein C-Induced Cardiomyopathy. Circ. Res. *123*, 1285–*129*7. https://doi.org/10.1161/circresaha.118.313089.
9. Huo, J.-L., Jiao, L., An, Q., Chen, X., Qi, Y., Wei, B., Zheng, Y., Shi, X., Gao, E., Liu, H.-M., et al. (2021). Myofibroblast Deficiency of LSD1 Alleviates TAC-Induced Heart Failure. Circ. Res. 129, 400–413. https://doi.org/10.1161/circresaha.120.318149.
10. Xiang, F.-L., Fang, M., and Yutzey, K.E. (2017). Loss of β-catenin in resident cardiac fibroblasts attenuates fibrosis induced by pressure overload in mice. Nat. Commun. 8, 712. https://doi.org/10.1038/s41467-017-00840-w.
11. Meng, Q., Yang, B., Qiao, Y., Wu, Y., Chen, J., Lin, X., and Molkentin, J.D. (2024). Genetic and Pharmacologic Inhibition of JAK1/2 Antagonizes Cardiac Fibrosis. Circulation *150*, 899–901. https://doi.org/10.1161/circulationaha.124.070340.

12. Hortells, L., Valiente-Alandi, I., Thomas, Z.M., Agnew, E.J., Schnell, D.J., York, A.J., Vagnozzi, R.J., Meyer, E.C., Molkentin, J.D., and Yutzey, K.E. (2020). A specialized population of Periostin-expressing cardiac fibroblasts contributes to postnatal cardiomyocyte maturation and innervation. Proc. Natl. Acad. Sci. 117, 21469–21479. https://doi.org/10.1073/pnas.2009119117.
13. Ock, S., Ham, W., Kang, C.W., Kang, H., Lee, W.S., and Kim, J. (2021). IGF-1 protects against angiotensin II-induced cardiac fibrosis by targeting αSMA. Cell Death Dis. 12, 688. https://doi.org/10.1038/s41419-021-03965-5.
14. Liu, H., Zhang, S., Xu, S., Koroleva, M., Small, E.M., and Jin, Z.G. (2019). Myofibroblast-specific YY1 promotes liver fibrosis. Biochem. Biophys. Res. Commun. *514*, 913–918. https://doi.org/10.1016/j.bbrc.2019.05.004.
15. Jung, S., Aliberti, J., Graemmel, P., Sunshine, M.J., Kreutzberg, G.W., Sher, A., and Littman, D.R. (2000). Analysis of Fractalkine Receptor CX3CR1 Function by Targeted Deletion and Green Fluorescent Protein Reporter Gene Insertion. Mol. Cell. Biol. 20, 4106–4114. https://doi.org/10.1128/mcb.20.11.4106-4114.2000.
16. Walter, W., Alonso-Herranz, L., Trappetti, V., Crespo, I., Ibberson, M., Cedenilla, M., Karaszewska, A., Núñez, V., Xenarios, I., Arroyo, A.G., et al. (2018). Deciphering the Dynamic Transcriptional and Post-transcriptional Networks of Macrophages in the Healthy Heart and after Myocardial Injury. Cell Rep. 23, 622–636. https://doi.org/10.1016/j.celrep.2018.03.029.
17. Heidt, T., Courties, G., Dutta, P., Sager, H.B., Sebas, M., Iwamoto, Y., Sun, Y., Silva, N.D., Panizzi, P., Laan, A.M. van der, et al. (2014). Differential Contribution of Monocytes to Heart Macrophages in Steady-State and After Myocardial Infarction. Circ. Res. *115*, 284–295. https://doi.org/10.1161/circresaha.115.303567.
18. Hulsmans, M., Clauss, S., Xiao, L., Aguirre, A.D., King, K.R., Hanley, A., Hucker, W.J., Wülfers, E.M., Seemann, G., Courties, G., et al. (2017). Macrophages Facilitate Electrical Conduction in the Heart. Cell *169*, 510-522.e20. https://doi.org/10.1016/j.cell.2017.03.050.
19. Pinto, A.R., Paolicelli, R., Salimova, E., Gospocic, J., Slonimsky, E., Bilbao-Cortes, D., Godwin, J.W., and Rosenthal, N.A. (2012). An Abundant Tissue Macrophage Population in the Adult Murine Heart with a Distinct Alternatively-Activated Macrophage Profile. PLoS ONE 7, e36814. https://doi.org/10.1371/journal.pone.0036814.
20. Molawi, K., Wolf, Y., Kandalla, P.K., Favret, J., Hagemeyer, N., Frenzel, K., Pinto, A.R., Klapproth, K., Henri, S., Malissen, B., et al. (2014). Progressive replacement of embryo-derived cardiac macrophages with age. J. Exp. Med. *211*, 2151–2158. https://doi.org/10.1084/jem.20140639.
